# The quarantine paradox: The economic cost of the increase in violence against women and girls in Sub-Saharan Africa

**DOI:** 10.3389/fpubh.2022.1029823

**Published:** 2022-10-25

**Authors:** Rediet Atalay, Girma Ayele, Saba Clarke, Miriam Michael

**Affiliations:** ^1^Internal Medicine, Howard University, Washington, DC, United States; ^2^Department of Economics, American University, Washington, DC, United States; ^3^Internal Medicine, University of Maryland, Baltimore, MD, United States

**Keywords:** violence against women, gender-based violence, intimate partner violence, COVID-19, sub-Saharan Africa, cost of violence

## Introduction

The worst global crisis since the Second World War, the COVID-19 pandemic, as of July 2022, has killed around 6.3 million people globally (1) and interrupted lives in countless ways. In response to the pandemic, public health officials implemented quarantine as a protective measure, while also leaving women and children to fell victim to increased violence: therein lies the paradox of the quarantine. Although “home quarantine” has proven an effective measure to fight pandemics since the fourteenth century, it has also created a pandemic of violence that dramatically increased the number of women abused by their intimate partners and the frequency of violent encounters. According to UN-Women (2), “as the pandemic raged on, the threat of a ‘shadow pandemic' of violence against women emerged.”

In addition to incurring heavy tolls for the victims, i.e., trauma and decreased quality of life, violence against women (VAW) also generates dire social and economic costs. The WHO argues that women suffer isolation, inability to work, lack of participation in regular activities, and failure to take care of themselves and their families due to intimate partners and sexual violence (3). The global cost of VAW is estimated to be 1.5 trillion dollars, which increased during the pandemic (4). The sub-Saharan African (SSA) region has the highest VAW prevalence worldwide, significantly worsening during the pandemic. This is alarming because VAW's social and economic burden further exacerbates the already high poverty rates in the region. In addition, women in these areas lack access to essential services, a plight that worsened during the lockdown. We will discuss the prevalence of VAW in the SSA region during COVID-19 lockdowns and propose future measures to address the root causes of the problem.

## Discussion

### Increasing trend of VAW during lockdown

**Figure 1:**
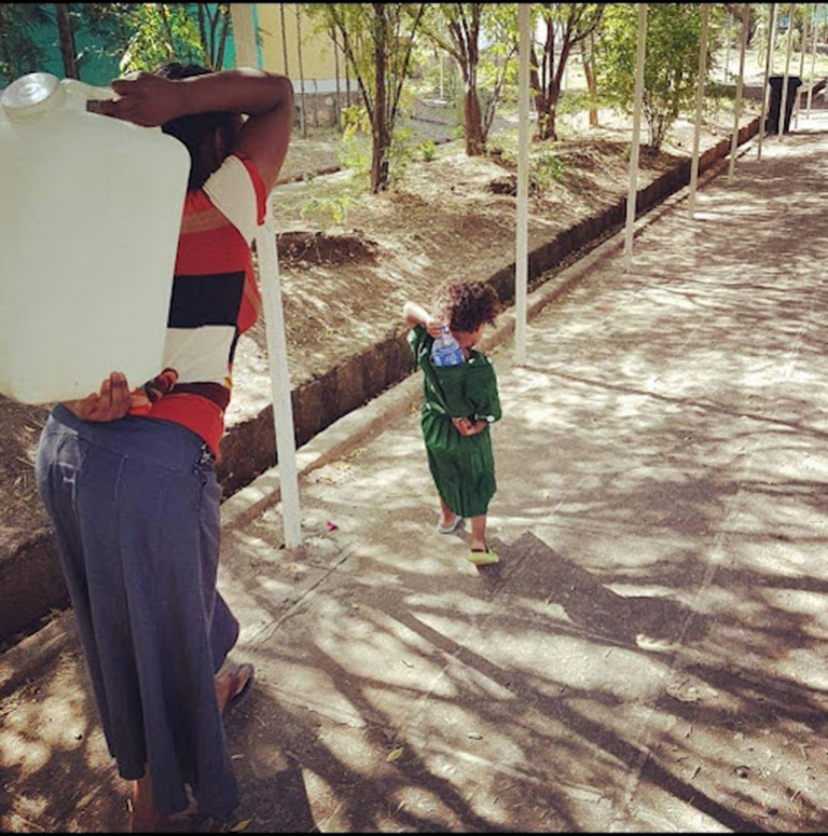
Little girl helping her mother fetch water showing the gender stereotyping in the community (March 8/2017, Ethiopia).

General factors that increase the likelihood of both intimate partner and sexual violence include low levels of education, a history of childhood abuse, witnessing family violence, antisocial personality disorder, harmful use of alcohol, harmful masculine behaviors (such as having multiple partners or attitudes that condone violence), community norms that privilege men, women's limited access to paid employment and the prevalence of gender inequality, e.g., discriminatory laws (3). The pandemic added with the community norm for female's role worsened the situation (Figure 1). Emergency situations, such as the COVID-19 pandemic, and exacerbates a preexisting problem of VAW.

VAW is often underreported: UN-Women estimates that less than 40% of victims come forward to report or seek help (4). The contributing factors for this underreporting include women's fear of worsening their situation, economic dependency on their abusers, shame, and lack of social solidarity and support.

However, the available data shows that global violence against women increased significantly compared to pre-pandemic figures. For example, Italy saw a 59% increase in calls to a hotline for gender-based violence (GBV) victims between 1 March and 16 April 2020 compared to the same period in 2019 (5). Similarly, Argentina witnessed the number of calls increase to 39%, and the country experienced 23 femicides in 33 days (6). The proportion of reported GBV in France, Cyprus, and Singapore has also seen an increase of 30, 30, and 33%, respectively, during the lockdown (4).

The prevalence of VAW by an intimate partner in the SSA region is 33% (3), the highest in the world. This situation significantly worsened during the period of lockdown. For example, the East African Community has reported a nearly 48% increase in VAW reported to the police or through toll-free lines. The Central African Republic reported increased injuries to women and children by 69% and a 27% increase in rape. The South African police recorded a 37% increase in GBV cases within the first week of the lockdown in April 2020. Similarly, VAW increased by 50% in Liberia during the first half of 2020. In Kenya, cases of rape and defilement also increased: more than 35% of all reported cases were related to these offenses (7). In Nigeria, the percentage of reported cases increased by 149%, a figure that only includes 23 out of 36 states (8).

### Cost of violence

The global cost of VAW has been estimated at least 1.5 trillion dollars (4). A World Bank (WB) study found that VAW costs 1.2–3.7% of gross domestic product (GDP) for some countries, and the WB emphasizes that these proportions of GDP are what many countries spend on primary education (9).

Empirical work undertaken by the International Monetary Fund (IMF) shows that GBV affects economic activity in SSA by significantly decreasing female employment (10). Additionally, as Vyas, (11) found a pay gap exists between abused and never-abused women in Tanzania. Tanzanian women experiencing violence earned 29% less than those not experiencing any abuse; this figure rises to 43% for women subject to harsher forms of violence. In SSA, VAW also adversely affects economic development, especially in countries without laws against GBV, in resource-rich countries where women enjoy less decision-making power, in countries with a high gender gap in education between partners, and during economic downturns (10).

Quantifying the direct impact of cost of violence remains a challenge for policymakers. Several studies used respondents' surveys, covering work absences, days of incapacity to undertake everyday activities, lost wages to the worker, and costs to the employer. The cost of violence is also quantified by using losses from injuries sustained in road traffic accidents, differentiated by injury level (12). The cost of health service utilization for the urgent treatment of injuries sustained during an assault by a partner has been approached in five different ways—first, victims' self-reporting of service utilization to representative surveys. Second, victims' self-reporting of injuries sustained to representative surveys modeled in combination with accident insurance data. Third, victims' reporting of injuries and acts of violence suffered to a national representative survey modeled through statistical methods predicting the likely course of treatment for specific injuries and their cost. Fourth, service utilization is established from administrative data employing diagnostic groups based on classifications of violence against the person. Fifth, service utilization is scaled from research reporting the prevalence and nature of intimate partner violence and assault injuries in accident and emergency department populations (12).

As the pandemic progressed, the virus's transmission increased in poor, crowded working conditions, especially in factories and the service sector, where women are overrepresented. That is, forty-two percent of the workforce is comprised of women while men constitute only thirty-two percent (13). The retail, tourism hotel, and manufacturing sectors were interrupted when the quarantine was instituted, which ended up distorting women's lives disproportionately. Workers in the informal economy, who are the most vulnerable, were also significantly affected by the lockdown. Globally, almost 1.6 billion informal economy workers are impacted during the lockdown, and 81% of informal jobs showed a decline in Africa (13).

Focusing on studies that estimate and calculate the economic toll of intimate partner violence (12) helps to draw attention to the fact that this was an essential factor in the financial losses perpetrated by the pandemic and pushes organizations and governments to intervene and make it part of the pandemic recovery plan to focus on mitigating violence against women.

### Measures to address the problem

To address the problem of VAW, it is imperative to gather more evidence about its magnitude and type and the circumstances under which VAW occurs. Such a data-driven approach would allow policymakers to understand the root cause of the problem and develop informed solutions that would support countries' efforts to combat violence. The WHO also endorses improving the methods for measuring VAW in monitoring the Sustainable Development Goals.

Community-based advocacy driven by VAW survivors helps create better access to other victims of violence. For women survivors of abuse, accessing informal support networks from friends and family and essential services—including psychosocial support—are crucial coping mechanisms. However, these support systems were not readily available during the lockdown in many countries, worsening VAW's social cost in Sub-Saharan Africa. In the future, policymakers should integrate protection and mitigation plans against VAW in emergencies like pandemics and natural disasters (3).

Providing economic support for women can also help combat Violence against women and can be undertaken by both governmental and non-governmental actors. Measures such as offering housing to women with financial insecurities, especially for victims who are financially dependent on their perpetrators (14), and direct cash transfers to women have been beneficial in many cases. For example, in Peru, a cash transfer program resulted in a 9% decrease in physical violence and an 11% decrease in emotional violence against women (9).

However, different types of economic support, including cash transfers, remain a short-term fix. To significantly reduce or eradicate VAW in Sub-Saharan Africa, we propose that policymakers improve law enforcement capacity, codify and implement anti-violence laws, and work to change social norms surrounding VAW, including the attitudes and behavior of males. Generally, it is essential to take the crisis as an opportunity to reconstruct policies and integrate laws that protect women in a post-COVID world.

Building improved institutional infrastructure, such as instituting a police task force that focuses mainly on preventing violence and helping abused women, is essential in the fight against VAW. Moreover, utilizing ICT and social media to ease the process of reporting abuse can be a powerful tool to reduce the frequency of violence. These measures can provide long-term solutions to combat VAW. Consequently, when a pandemic or crisis occurs in the future, Sub-Saharan African countries will benefit from a more resilient infrastructure and coping mechanisms that can minimize VAW's social and economic costs.

## Conclusion

This article reviewed the significant increase of VAW during the COVID-19 pandemic and its economic cost in sub-Saharan Africa. In a region with one of the highest incidences of Gender-based violence, addressing the issue is hampered by the lack of robust data and studies that address and quantify the monetary loss VAW has on the economy. With a lack of standardization in data collection and even less on calculating fiscal impact solutions that are put in place, the efficacy of the interventions. The interventions by governments and organizations are imperative to tackle the problem. Still, there needs to be support for this effort to gather data on incidence and prevalence and synthesize the data into measurable metrics that can be quantified and reproduced.

## Author contributions

The authors confirm their contribution to the paper as follows. The authors worked equally on the paper. RA worked on the abstract, conclusion, and some parts of the discussion. GA worked on the discussion. MM worked on the discussion and introduction. SC worked on the discussion and final editing. All authors contributed to the article and approved the submitted version.

## Conflict of interest

The authors declare that the research was conducted in the absence of any commercial or financial relationships that could be construed as a potential conflict of interest.

## Publisher's note

All claims expressed in this article are solely those of the authors and do not necessarily represent those of their affiliated organizations, or those of the publisher, the editors and the reviewers. Any product that may be evaluated in this article, or claim that may be made by its manufacturer, is not guaranteed or endorsed by the publisher.
